# The impact of an amino acid-humus preparation on lawn boning value

**DOI:** 10.1038/s41598-025-90862-y

**Published:** 2025-02-24

**Authors:** Adam Radkowski, Iwona Radkowska, Karen Khachatryan, Michał Kozdęba, Henryk Bujak, Karol Wolski

**Affiliations:** 1 Department of Agroecology and Plant Production, University of Agriculture in Kraków, Mickiewicza 21, 31-120 Kraków, Poland; 2 Department of Cattle Breeding, National Research Institute of Animal Production, Krakowska 1, 32-083 Balice, Poland; 3 Laboratory of Nanomaterials and Nanotechnology, Faculty of Food Technology, University of Agriculture in Krakow, Balicka 122, 30-149 Krakow, Poland; 4 Department of Production and Power Engineering, University of Agriculture in Krakow, Ul. Balicka 116 B, 30-149 Krakow, Poland; 5 Department of Genetics, Plant Breeding and Seed Production, Wrocław University of Environmental and Life Sciences, Grunwaldzki 24A, 53-363 Wrocław, Poland; 6 Research Center for Cultivar Testing (COBORU), 63-022 Słupia Wielka, Poland; 7 Department of Agroecology and Plant Production, Wrocław University of Environmental and Life Sciences, Grunwaldzki 24A, 50-363 Wrocław, Poland

**Keywords:** Lawn, Amino acids, Humic acids, Vegetation indices, Natural variation in plants, Plant development, Plant ecology, Plant stress responses

## Abstract

The application of biostimulants in fertilisation has been shown to enhance plant metabolism, stimulate physiological processes, and mitigate adverse environmental and pathogenic stresses. This study, conducted at the Agricultural University of Krakow (2021–2023), evaluated the efficacy of a foliar amino acid preparation enriched with humic acids (L-Amino + ® Humus) on turf quality. Four experimental variants were established: an untreated control (Variant I) and three treated plots sprayed with the biostimulant at 1.0 (Variant II), 2.0 (Variant III), and 3.0 L ha^−1^ (Variant IV). Higher application rates (Variants III and IV) significantly improved aesthetic turf quality and reduced fungal disease incidence. Compared to the control, Variant IV (3.0 L ha^−1^) decreased snow mould (*Microdochium nivale*) infections by 8% and brown spot (*Rhizoctonia solani*) prevalence by 12%. Variant III (2.0 L ha^−1^) also demonstrated positive effects, with 6% and 8% reductions in snow mould and brown spot, respectively. Furthermore, the Normalized Difference Vegetation Index (NDVI) and leaf greenness index (SPAD) chlorophyll meter values increased by 6% and 8%, respectively, in Variant IV relative to the control, indicating enhanced leaf greenness and photosynthetic efficiency. Nutrient analysis revealed that Variants III and IV significantly elevated macro- and micronutrient concentrations (e.g., nitrogen, potassium, iron) in plant biomass compared to the control. In contrast, Variant II (1.0 L ha^−1^) showed no statistically significant improvements in nutrient content or disease resistance. These findings suggest that L-Amino + ® Humus at doses of 2.0–3.0 L ha^−1^ enhances turf quality, bolsters resistance to fungal pathogens, and improves nutrient assimilation. Lower doses (1.0 L ha^−1^), however, were ineffective under the tested conditions. The study underscores the dose-dependent efficacy of biostimulants in sustainable turf management.

**Supplementary Information:**

The online version contains supplementary material available at 10.1038/s41598-025-90862-y.

## Introduction

The presence of lawns in urban environments is significant, serving not only an aesthetic purpose but also a crucial role in promoting public health through encouraging physical activity and enhancing the mental well-being of the general public^1^. In order to perform these functions effectively, lawns—especially those of a utilitarian nature – require appropriate care and balanced fertilisation in order to ensure their quality and longevity^2^. The growth and aboveground phytomass production of lawns is strongly influenced by a number of environmental and agronomic factors, with soil type and quality playing a particularly important role. Soil characteristics such as texture, structure, pH, organic matter content, nutrient availability and water holding capacity have a significant effect on nutrient uptake and overall grass health. These conditions, combined with other factors such as fertiliser regime, growing season, irrigation, intensity of use and species composition of the grass mixture, determine the overall productivity and quality of the lawn^3,4^.

Intensive, long-term mineral fertilisation, especially with nitrogen fertilisers, can increase soil acidity, disturb the nutrient balance, reduce crop quality^5,6^, reduce the humic acid content of the soil^7^ or reduce the species diversity of beneficial soil microorganisms, which are crucial for maintaining soil fertility. Biostimulants may offer an alternative to chemical inputs in agricultural and horticultural production. A variety of natural compounds and extracts, containing bioactive components, can act as biostimulants. The typical ingredients of a biostimulant include humic, fulvic and salicylic acids, minerals, amino acids, chitosan, vitamins, as well as poly- and oligosaccharides, which exhibit a range of biostimulant properties^8,9^. In response to high demands and environmental concerns, the use of biostimulants can be an effective solution. The application of biostimulants has been demonstrated to exert a beneficial influence on the metabolic processes of lawns. They stimulate vital processes and mitigate the effects of adverse environmental conditions (drought, salinity, temperature fluctuations) and pathogens^10,11^. Biostimulants, such as amino acids and humic acids, have the potential not only to stimulate plant growth, but also to reduce the negative effects of environmental stresses such as salt, drought, temperature fluctuations and pathogens^5–7^.

Amino acids such as glutamate, histidine, proline, betaine and glycine, and humic acids, which are natural components of organic matter derived from plant, animal and microbial decomposition, have been shown to activate plant metabolic pathways^12,13^. They are also crucial for plant defence against environmental factors^14,15^. Humic acids, an essential component of soil humus, are also used as biostimulants. They can be applied directly to the soil or by foliar spray. Humic acids can promote the proliferation of beneficial soil microorganisms^16^, increase the nutrient content of the soil^17^, which can be more easily taken up by the plant root system, and increase plant resistance to stress factors, pests and fungal diseases^18^.

The available evidence from scientific studies indicates that preparations based on these substances can have a marked effect on nutrient uptake, nitrogen and iron metabolism, and overall plant performance^19^.

Despite the relatively large number of publications on the use of biostimulants in plantations^20–23^, the scientific information available on their optimal use remains incomplete. It is hypothesised that biostimulants can serve as a valuable source of nutrients, including nitrogen, for lawns^24^. The experiment’s authors posit that high-quality lawns can be achieved through repeated foliar application of nutrients present in biostimulants, negating the necessity for intensive mineral fertilisation^25,26^.

The objective of this study is to investigate the effects of amino acid humus biostimulants on key lawn quality parameters, including leaf area index (LAI), chlorophyll content (SPAD values), greenness index (NDVI) and mineral uptake efficiency, which are key indicators of both aesthetic and functional lawn quality.

Despite the growing number of studies on biostimulants, their specific effects on parameters such as LAI, chlorophyll content, greenness index (NDVI) and nutrient uptake in lawns are still poorly understood^23,27–30^. The present study aims to fill this gap by investigating the effect of a new amino acid humus preparation (L-Amino+^®^ Humus) in a long-term lawn trial. This is the first time that this product has been tested. By evaluating visual characteristics (overall appearance, canopy, leaf structure, leaf colour) and functional parameters (NDVI, SPAD, LAI values), as well as macro- and micronutrient content, this study aims to provide practical guidance on the optimal use of biostimulants in lawn care. The novelty of this study lies in the comprehensive evaluation of the effect of the tested biostimulant on both aesthetic and functional lawn parameters.

## Materials and methods

### Study site

The experiment was conducted between 2021 and 2023 at the experimental station of the University of Agriculture in Krakow (50° 07′ N, 20° [05′ E) (Poland). The study site comprised degraded chernozem, more precisely Haplic Phaeozems (Siltic), formed from loess. Prior to the initiation of the experimental procedure, the soil exhibited an alkaline reaction (pH_KCl), while the content of total nitrogen (N), available phosphorus (P), available potassium (K), and organic matter (humus) was at a medium level, whereas the magnesium (Mg) content was high. The chemical properties of the soils are presented in Table 1. Prior to the initiation of the experiment, soil samples were collected and subsequently evaluated in accordance with established protocols^31^.

**Table 1:** Chemical properties of soil in the study site.

Parameter/Element	Amount	Level/range
pH_KCl_	7.6	Alkaline
N (total nitrogen)	2.00 g kg^−1^ soil	Medium
P (available phosphorus)	63.22 mg kg^−1^ soil	Medium
K (available potassium)	180.21 mg kg^−1^ soil	Medium
Mg (magnesium)	42.16 mg kg^−1^ soil	High
Organic matter (humus)	35.0 g kg^−1^ soil	Medium

### Experiment design and pratotechnical description

The experiment was conducted in accordance with the agrotechnical recommendations for the establishment of lawns^32^. The grass mixture employed in the study was Super Trawnik, provided by Planta Sp. z o.o., Tarnów, Poland (Table 2).

**Table 2:** Composition of evaluated grass mixture.

Grass species	Variety	Share in grass mixture (%)
Perennial Ryegrass (*Lolium perenne* L.)	Stadion	10
Perennial Ryegrass (*Lolium perenne* L.)	Bokser	55
Tall Fescue (*Festuca arundinacea* Shreb.)	Escalante	10
Red Fescue (*Festuca rubra* L.)	Gross	6
Red Fescue (*Festuca rubra* L.)	Adio	19

The 10 m^2^ plots were sown with a grass mixture at a rate of 26.0 g per m^2^. Sowing started on 7 April 2021 and the experiment was completed on 25 October 2023. A pre-sowing application of 40 kg N/ha, 33 kg P and 60 kg K was made, while a post-harvest application of 65 kg N and 60 kg K/ha was made. In the subsequent years of full cultivation, 190 kg N, 35.2 kg P and 124.5 kg K/ha were applied. The fertiliser doses used are in accordance with the COBORU method^33,34^. The nitrogen fertilisers were applied as 34% ammonium nitrate, the phosphorus fertilisers as granular triple superphosphate (20.2% P), and the potassium fertilisers as potassium salt (49.8% K). During the growing season, the grass was mowed 11–12 times (when it reached a height of 8 cm) to a height of 4 cm according to COBORU recommendations for ‘relax’ type mixtures^32,35^.

The experiment was conducted in accordance with a randomised block design, with three replicates at each of four sites. The location and appearance of the experiment is shown in Figure S1 (see Supplementary material). The control site was not treated (Variant I), while the remaining three sites were sprayed with L-Amino+^®^ Humus at varying rates of 1.0 (Variant II), 2.0 (Variant III), and 3.0 L ha^−1^ (Variant IV). The tested preparation, L-Amino + Humus, is produced by the Agro-sorb Company in Częstochowa, Poland. L-Amino + Humus is an organic growth biostimulator. The preparation contains biologically active, naturally occurring, free L-α amino acids, formed by enzymatic hydrolysis. In addition to biologically active free L-α amino acids, L-AMINO + Humus contains sterols and lipid compounds. The formulation is composed of the following: free amino acids (52 g/L, 5% m/m), total amino acids (10%, m/m), total nitrogen (N) (1.6%, m/m), organic carbon (C_org_) (3.5%, m/m), organic matter in dry matter (65%, m/m), humic acids (2.0%). The list of free amino acids present in the formulation is as follows: aspartic acid (0.225%), serine (0.1605%), glutamic acid (0.907%), glycine (1.3715%), histidine (0.104%), arginine (0.0655%), threonine (0.1615%), alanine (0.262%). The remaining amino acids present in the formulation are as follows: proline (0.1735%), cysteine (0.2175%), tyrosine (0.087%), valine (0.2755%), methionine (0.1745%), lysine (0.154%), isoleucine (0.09%), leucine (0.09%), phenylalanine (0.109%), tryptophan (0.109%) and tryptamine (0.025%). The formulation is presented in liquid solution.

The product was applied as a foliar spray three times during the growing season, in the first ten days of April, July and October. Except in the year of sowing when the first application was made on 11 May and subsequent applications as above.

### Weather conditions

The weather conditions during the aforementioned experiment demonstrated notable variability. The meteorological data were obtained from a meteorological station (WS-GP2 series meteorological station, Geomor-Technik, Szczecin, Poland). The total precipitation and mean air temperature during the growing season (April to September) for 2021, 2022, and 2023 are presented in Table 3. The years 2021–2023 at the Experimental Station in Prusy, affiliated with the University of Agriculture in Kraków, exhibited distinct meteorological conditions (Table 3). 2021 was characterised by exceptionally high precipitation, particularly during the summer months, culminating in the highest annual total rainfall of the three-year period. In contrast, 2022 saw significantly drier conditions, with below-average precipitation levels, most notably during spring and summer. 2023, however, demonstrated a more balanced precipitation distribution, marked by elevated rainfall at the start of the year and moderate summer values.

Regarding temperature, 2021 was comparatively cooler, especially in winter, whereas 2022 and 2023 experienced warmer conditions, particularly during spring and summer. This trend suggests a gradual shift toward milder temperatures in the latter two years.

**Table 3:** The data set comprises the total precipitation and mean air temperature at the experimental station in Prusy, university of agriculture in Kraków, for the years 2021–2023.

Month	Precipitation (mm)			Average temperature (°C)		
	2021	2022	2023	2021	2022	2023
January	31.6	21.6	66.2	-0.9	0.4	2.8
February	40.2	24.4	35.8	-0.9	3.4	1.3
March	19.8	14.6	19.1	3.6	4.0	5.7
April	64.0	41.0	53.8	6.4	7.1	7.9
May	86.8	20.6	90.0	12.6	15.2	13.0
June	112.4	35.2	65.8	19.5	19.7	17.8
July	139.2	85.8	106.0	21.3	19.6	20.2
August	191.0	65.2	97.6	17.5	20.6	20.3
September	39.6	51.8	55.0	14.4	12.9	14.4
October	22.0	17.4	57.5	9.4	11.8	8.7
November	43.0	45.0	85.5	4.9	-2.4	3.1
December	17.8	23.5	12.0	-0.7	-3.9	-1.1
Total precipitation IV-IX	633.0	299.6	468.2	–	–	
Total precipitation I-XII	807.4	446.1	744.3	–	–	
Average temperature IV-IX	–	–	–	15.3	15.8	15.6
Average temperature I - XII	–	–	–	8.9	9.0	9.5

In the event of prolonged drought conditions, defined by the presence of dry soil to a depth of 3 cm and the inability of plants to lift when pressed with the hand, the application of regular irrigation was undertaken via sprinkling at three-day intervals. The volume of water applied per watering was approximately 10 L m^− 2^.

### Methods for the assessment of plant quality

The assessment of the use value of the turf was conducted in accordance with the methodology developed by the Committee on Sports Grounds (COBORU) for turf grasses^36,37^. The use value of the lawn was assessed three times during the growing season: spring (mid-May), summer (mid-July) and autumn (mid-October), approximately 14 days after application of the tested products. Except in the year of sowing, when the first evaluation was made on 25 May, and the others as above. The assessment was conducted visually and included qualitative characteristics, which were rated on a nine-point scale (1 = poor characteristic, 9 = excellent characteristic)^33^. The assessment of the lawn encompassed a range of characteristics, including its overall aesthetic appearance, turfing, leaf colour and structure in autumn, as well as disease tolerance during periods of disease severity. The identification of fungal species was conducted in accordance with phytopathological keys and monographic studies^38–44^.

The impact of the applied factors on chlorophyll content was evaluated for each year of the study. The leaf greenness index (SPAD) was determined using a Minolta SPAD 502DL chlorophyll meter (Minolta, Osaka, Japan), with measurements taken on the upper leaves. The device measures the difference in light absorption by the leaf at wavelengths of 650 and 940 nm, and the ratio of these values represents the leaf greenness index, or chlorophyll content. Measurements were taken in each plot, on a total of 30 fully expanded leaves. The leaf area index (LAI) was quantified utilising the SunScan Canopy Analysis System (Delta-T Devices Ltd, UK), while the Normalized Difference Vegetation Index (NDVI) was determined employing the GreenSeeker Handheld Optical Sensor Unit (NTech Industries, Inc, USA). For each variety, 12 samples for LAI and 4 samples for NDVI were taken in each season studied (spring, summer, autumn). Over three years, this gives 108 samples for LAI and 36 samples for NDVI for each variant. It has been demonstrated that nitrogen is associated with NDVI and SPAD values, given its direct influence on chlorophyll content, leaf area development, and overall plant biomass. These factors are pivotal in determining these indices. Mineral content was determined using the Weende method^31^.

### Statistical analysis

The collected results were subjected to statistical processing using the Statistica software (version 13.0, StatSoft, Kraków, Poland, https://www.statsoft.pl/). The data were presented in box plots, which illustrate the median, the range of the most frequent results (between the first and third quartiles), the range of non-frequent values (away from the most frequent by no more than one and a half quartile values of the quartile range), and outliers. In order to ascertain the statistical significance of the observed differences between the means of the indicators, a one-way and two-way ANOVA were conducted, employing the Tukey-Kramer HSD test with a significance level of *p* = 0.05. In the two-way ANOVA, the dose rate was designated as the primary factor, with the year in which the study was conducted serving as the secondary factor. The initial null hypothesis was that no significant differences existed between the groups (i.e., between the various doses and between the different years). The subsequent null hypothesis was that no significant relationship existed between the dose used and the year of use. In the case of indicators that did not exhibit notable differences in graphical representation, a corrected true difference (Ad’) measure was conducted.

Given the considerable resemblance in the graphical representation of the changes in the NDVI, LAI and SPAD indices, it was deemed appropriate to calculate the corrected mean difference index in accordance with the following formula:

1 $$\:{A}_{d}^{{\prime\:}}=\frac{{\sum\:}_{k=1}^{n}({a}_{n}-{b}_{n})}{n}\cdot\:\frac{|{\sum\:}_{k=1}^{n}\left({a}_{n}-{b}_{n}\right)|}{{\sum\:}_{k=1}^{n}|{a}_{n}-{b}_{n}|}$$

In the case of two quantities, a_n_ and b_n_, where one always assumes a larger value than the other, the indicator A_d_’ is equivalent to the mean difference. In the alternative scenario, the mean is multiplied by the coefficient of variation. The closer the coefficient of variation is to zero, the more frequently the leading magnitude changes. This results in an improved mean difference ratio.

## Results

This chapter presents the results of a statistical analysis of the changes in the indicators discussed in Chap. 2.4 and in the levels of selected minerals. The application of L-Amino + Humus at doses of 2 and 3 L ha^−1^ (variants III and IV) resulted in higher visual evaluation indices, NDVI, LAI and SPAD compared to the control sample (variant I). Furthermore, in variants III and IV notable increases were observed in the concentration levels of selected mineral nutrients.

In order to examine the dose rate relationship, the effect of the different years and the relationship between them on the results of the study, a two-way ANOVA was carried out. The results of this analysis are presented below.

All the indicators tested yielded a p-value that was significantly less than 0.05 in the initial tests for the factor “dose size,” thereby allowing us to reject the null hypothesis and conclude that there is a statistically significant difference between the doses utilized (Tables 4 and 5). Moreover, the majority of indicators yielded the same result for the factor of ‘year’. Only a single indicators achieved a p-value below the 0.05 threshold in this instance. The aim of the present work is to study the effect of the dose size of the formulation on the values of the indicators considered. However, in relation to the above, in subsection 3.4 we will also outline the possible influence of the second factor, i.e. the year.

**Table 4:** p-values for dose (factor A), year of study (factor B) and interaction between them in two-way ANOVA for visual assessment, NDVI, LAI and SPAD indices.

	General aspect	Turf density	Leaf colour	Leaf texture	Pink snow mold	Leaf spot	Stem rust	NDVI	LAI	SPAD
Factor A	0*	0*	0*	0*	0*	0*	0*	0*	0*	0*
Factor B	0*	0.162	0*	0.021	0.059	0*	0*	0*	0.261	0*
Interaction AB	0.196	0.912	0.102	0.101	0.450	0.294	0.502	0.911	0.999	0.999

0* - means the result is significantly less than 0.001.

**Table 5:** p-values for dose (factor A), year of study (factor B) and interaction between them in two-way ANOVA for mineral components.

	*P*	K	Ca	Mg	Na	Mn	Fe	Zn	Cu
Factor A	0*	0.004	0.002	0*	0*	0*	0*	0*	0*
Factor B	0.024	0*	0.035	0.248	0.639	0.020	0.014	0.170	0.949
Interaction AB	0.982	0.998	0.975	1.000	0.996	0.996	1.000	0.898	0.973

0* - means the result is significantly less than 0.001.

In the second-stage test, all indicators yielded values that were significantly above 0.05, thereby precluding the rejection of the null hypothesis. In light of the aforementioned considerations, it was deemed prudent to forego further post-hoc analysis and dependency graph analysis, given the probable absence of a significant correlation between the applied dose and the year of application.

### Visual assessment

The index of the general aspect, namely the appearance of the turf and its attractiveness, exhibited a range of 4.8 to 9.0, contingent on the applied fertilisation rate and the year of the study (Supplementary material, Figure S2). The impact of fertilisation on the aesthetic value of the turf was evident from the outset of the study, with values ranging from 4.8 to 8.4 in the first year. In the second year of use, values ranged from 5.2 to 8.9, while in the third year, they ranged from 7.0 to 9.0. In terms of seasonal variation, the highest values were recorded in autumn (with an average of 8.6 across the three-year study period), followed by spring (8.2) and then summer (7.0). The application of fertiliser treatments resulted in a significant differentiation in the overall aspect. The mean value for the three-year study period was 7.3 for the control site, 7.7 for the first treatment (1 L ha^−1^), 8.2 for the second treatment (2 L ha^−1^) and 8.5 for the third treatment (3 L ha^−1^) (Supplementary material, Figure S3).

A further characteristic examined was the density of the lawn canopy that covered the ground during the growing season. A higher score was awarded in instances where a greater proportion of the soil was covered by leaves. This trait exhibited a range of 4.7 to 9.0. The mean score for the control sample during the study period was 6.9, for the first treatment 8.2, for the second treatment 8.1 and for the third treatment 8.7.

The highest value for leaf colour was observed in the third treatment, which had the highest application rate. The mean value for this trait over the three-year period was 8.5, while the lowest value, 7.1, was recorded in the control sample (Supplementary material, Figure S2). Another trait analysed was that of leaf structure, with values ranging from 4.4 to 9.0 being observed. The highest value was recorded in the third treatment (3 L ha^−1^).

With regard to susceptibility to snow mould (*Microdochium nivale*), values ranged from 5.2 to 9.0 across the various sites (Supplementary material, Figure S2). On the scale employed, a rating of 9 indicates the absence of any symptoms of infestation, whereas a rating of 1 signifies that the plants are entirely infested^6,33^. A comparable pattern was evident in the susceptibility to brown leaf spot caused by *Drechslera siccans*, with values fluctuating between 5.1 and 9.0. In both instances, the highest values were observed in the third treatment. With regard to stem rust (*Puccinia graminis*,* Puccinia festuce*), the values observed ranged from 3.5 to 9.0 (Supplementary material, Figure S3). Similarly, higher doses of the tested preparation yielded a lower incidence of infestation by this pathogen.

In order to illustrate the results more clearly, the mean values of the indices obtained and the ranges containing the most frequent values, excluding the extreme percentiles, were analysed. Table 6 shows the mean values of the indicators over the three-year study period, together with the minimum ranges that include the 10th and 90th percentiles. In addition, a one-way ANOVA was performed on the individual indices. This allowed groups of data with statistically significant differences to be identified.

**Table 6:** Visual assessment coefficient values for 2021–2023.

	Variant I	Variant II	Variant III	Variant IV
General aspect	7.27 ± 2.00^a^	7.73 ± 2.20^ab^	8.16 ± 1.50^bc^	8.51 ± 0.43^c^
Turf density	6.88 ± 2.15^a^	8.22 ± 0.99^bc^	8.08 ± 1.59^b^	8.75 ± 0.46^c^
Leaf colour	7.07 ± 1.59^a^	7.73 ± 1.71^b^	8.25 ± 1.74^bc^	8.46 ± 0.86^c^
Leaf texture - slenderness	6.67 ± 1.96^a^	7.47 ± 1.46^b^	7.84 ± 1.37^b^	7.94 ± 1.52^b^
Pink snow mold	8.43 ± 1.55^a^	8.38 ± 1.85^b^	8.52 ± 1.72^b^	8.91 ± 0.09^b^
Leaf spot	7.35 ± 2.07^a^	7.90 ± 1.50^a^	8.68 ± 1.01^b^	8.97 ± 0.03^b^
Stem rust	7.30 ± 2.70^a^	7.87 ± 2.45^ab^	8.45 ± 1.29^bc^	8.80 ± 0.65^c^

The same superscript letters in each row demonstrate a lack of significant difference between values (*p* < 0.05).

It is evident that the average values for Variants II, III and IV were significantly higher than those observed in the control sample, and that the range of values was also smaller. To illustrate, in Variant IV, the General aspect indicator attained values within the range of 8.08 to 8.94 (omitting the 10% smallest and largest results), whereas in the control sample, it reached values within the range of 5.27 to 9.00. Table 4 also indicates that there were significant differences in values for all indicators between Variants III and IV and the control sample (Variant I).

### NDVI, LAI and SPAD indices

**Fig. 1:**
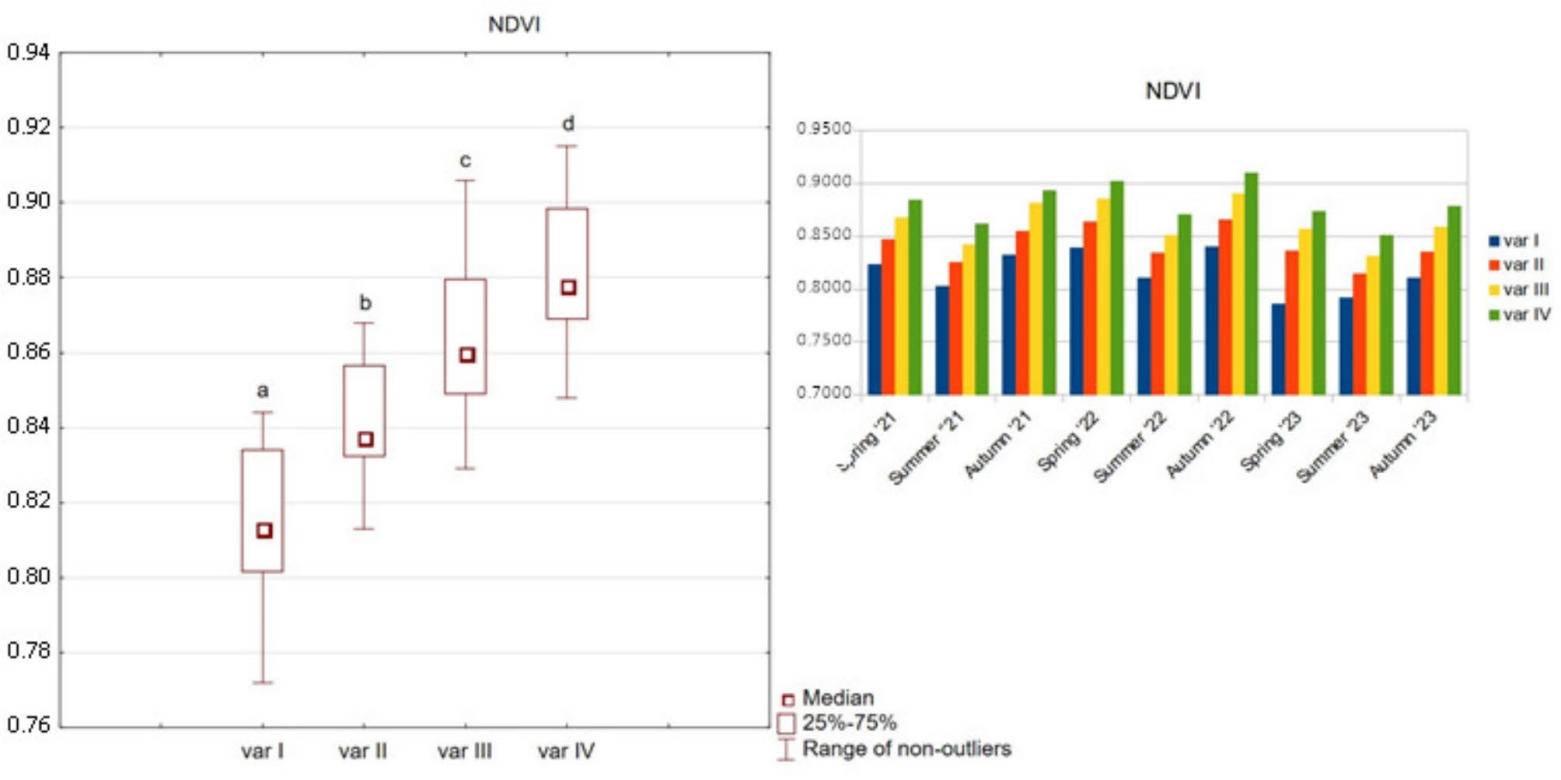
NDVI index for options variants I, II, III, and IV.

The greenness index (NDVI) showed a slight difference between the examined treatments (variants), with values ranging from 0.772 to 0.915. Variant IV (3 L ha^−1^) was characterised by a significantly higher value of this index compared to variants II and I. Over the entire study period, statistically significant differences in the achieved values of this indicator are noticeable (Fig. 1).

**Fig. 2:**
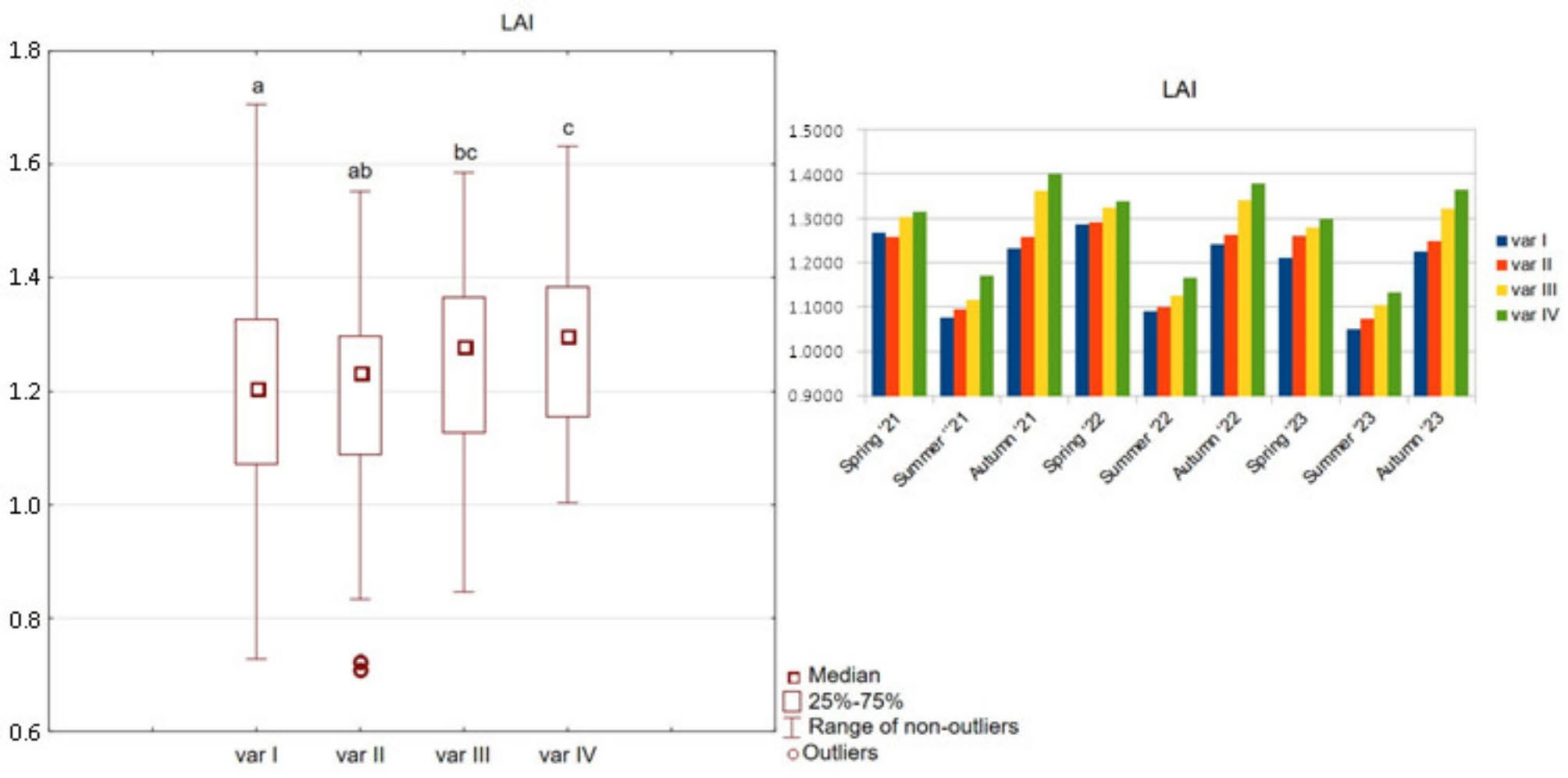
LAI index for options variants I, II, III, and IV.

The leaf area index (LAI) exhibited a range of 0.7 to 1.7. The observed differences were confirmed statistically, depending on the treatment and study years (Fig. 2). In particular, variant IV exhibited a more expansive assimilative area within the grassland ecosystem.

**Fig. 3:**
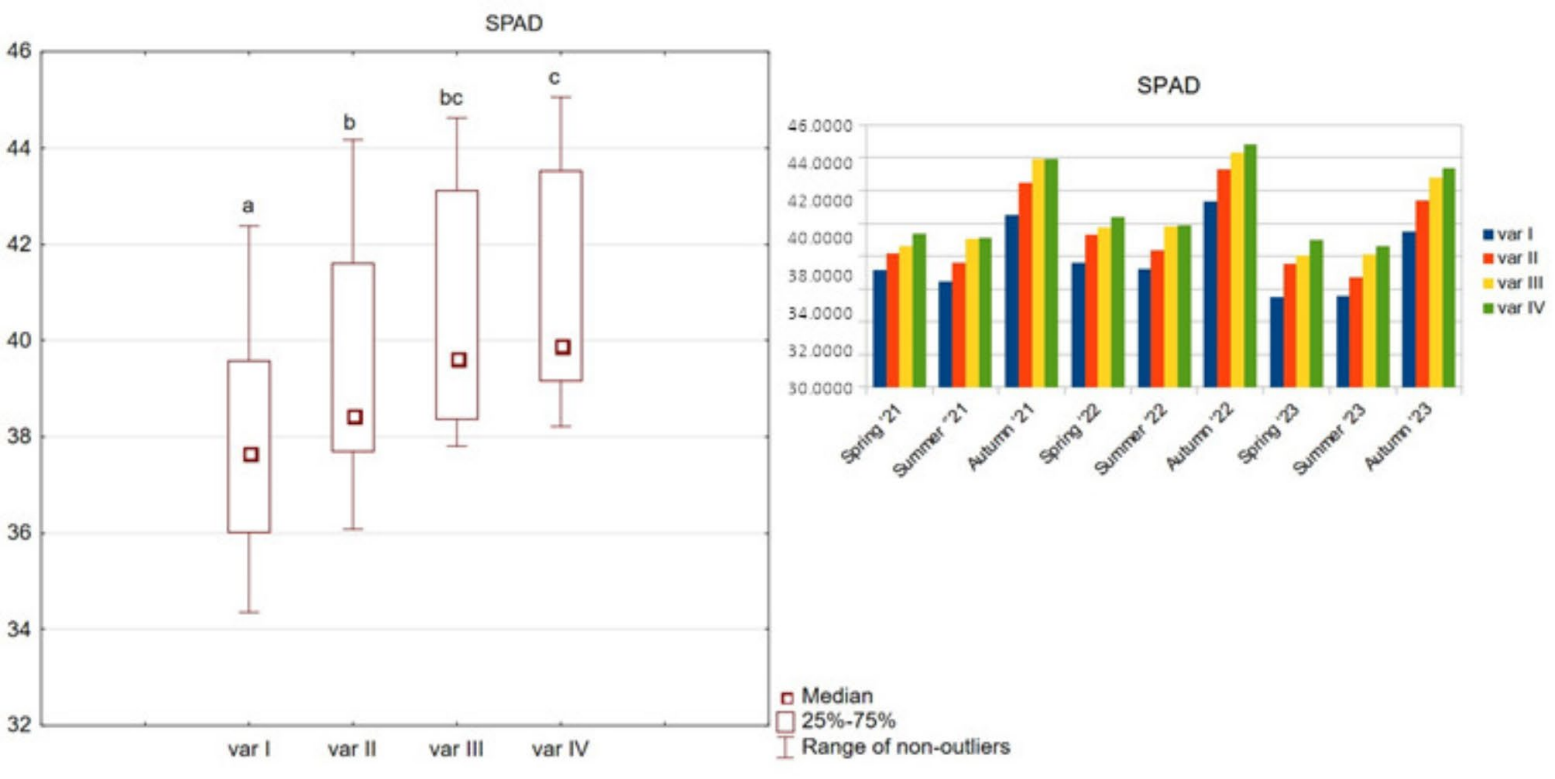
SPAD index for options variants I, II, III, and IV.

The mean values of the leaf greenness index (SPAD) at the various test dates exhibited a range from 34.34 to 45.06. The object exhibiting the highest fertilisation rate, averaged over the test period, demonstrated an 8% higher value of this index in comparison to plants from the control object. The most statistically significant differences from the control sample were once again observed for variants III and IV (Fig. 3).

Given the resemblance in the graphical representation of the NDVI, LAI and SPAD indicators, a corrected mean difference index (A_d_’) was also calculated. The relative and absolute differences between variants II, III, IV and variant I were subjected to examination. The A_d_’ values for the LAI index yielded results of less than 3% for variants II and III, thereby indicating minimal discrepancies from the control sample (Table 7). Only variant IV demonstrated a statistically significant difference of 4.82%. In contrast, the NDVI and SPAD indices demonstrate unambiguous increases across all variants. An increase of 3.26% and 4.11% was observed for variant II, while variant III exhibited an increase of 5.82% and 6.91%. Variant IV demonstrated an increase of 8.01% and 8.11%. It is noteworthy that in the case of NDVI and SPAD, Ad’ reached the same values as the classic mean difference, which serves to prove the significant influence of the tested preparation used in variants II, III, and IV on these indices.

**Table 7:** A_d_’ ratio between options II, III, IV and option I.

Variant	Variant II	Variant III	Variant IV
LAI	0.002	0.024	0.057
LAI (%)	0.17%	2.05%	4.82%
NDVI	0.027	0.047	0.065
NDVI (%)	3.26%	5.82%	8.01%
SPAD	1.557	2.615	3.073
SPAD (%)	4.11%	6.91%	8.11%

### Mineral components

Table 6 illustrates the impact of foliar fertilisation on the concentration of macronutrients and micronutrients in plants. Similar to Table 4, here too the averages of 36 samples were compared together with the minimum ranges, which include the 10th and 90th percentiles. Here, a one-way ANOVA was also performed and groups of data with statistically significant differences were identified. The study demonstrated that the application of L-Amino + Humus in variants III and IV led to a notable enhancement in the concentration of macronutrients in comparison to the control sample. With regard to phosphorus (P), the content of this element in plants ranged from 1.86 to 2.71 g kg^−1^ d.m (Supplementary material, Figure S4). A statistically significant increase in phosphorus content was observed following the application of fertilisation at a dose of 3 L ha^−1^ (Table 8). Similar outcomes were observed for potassium, with a range of 34.79 to 41.81 g kg^−1^ DM, and magnesium (1.53 to 2.46 g kg^−1^ DM), where a dose of 3 L ha^−1^ also resulted in a statistically significant increase in the content of these elements. In contrast, foliar fertilisation with L-Amino + Humus had no statistically significant effect on calcium (Ca) and sodium (Na) content, which ranged from 2.61 to 3.44 g kg^−1^ DM and 0.08 to 0.14 g kg^−1^ DM, respectively (Supplementary material, Figure S4). With regard to micronutrients, it was determined that fertilisation at a rate of 1 L ha^−1^ (variant II) did not exert a statistically significant influence on the concentration of these elements in plant biomass (Table 6). The majority of measurements in the control sample indicated manganese levels of 131.42-147.49 mg kg^−1^ DM, iron levels of 257.46-354.69 mg kg^−1^ DM, zinc levels of 54.79–72.05 mg kg^−1^ DM, and copper levels of 9.55–11.73 mg kg^−1^ DM( (Supplementary material, Figure S5). Variant II exhibited comparable levels of these elements, albeit with a greater range of manganese values (122.17-167.25 mg kg^−1^ DM). In contrast, the application of fertiliser at rates of 2 L ha^−1^ and 3 L ha^−1^ (Variants III and IV) resulted in statistically higher levels of all micronutrients tested (Table 8). The majority of measurements in Variant IV indicated manganese levels of 138.74-160.64 mg kg^−1^ DM, iron levels of 298.85-381.96 mg kg^−1^ DM, zinc levels of 66.53–78.55 mg kg^−1^ DM and copper levels of 10.80-13.54 mg kg^−1^ DM.

**Table 8:** The mineral coefficient values for the period spanning 2021 to 2023.

	Variant I	Variant II	Variant III	Variant IV
P (g/kg DM)	2.05 ± 0.35^a^	2.23 ± 0.39^b^	2.34 ± 0.43^bc^	2.46 ± 0.42^c^
K (g/kg DM)	37.61 ± 3.51^a^	38.27 ± 4.20^ab^	38.88 ±3.54^ab^	39.83 ± 3.34^b^
Ca (g/kg DM)	2.84 ± 0.40^a^	2.97 ± 0.61^ab^	2.99 ± 0.41^ab^	3.17 ± 0.51^b^
Mg (g/kg DM)	1.70 ± 0.42^a^	1.89 ± 0.51^ab^	1.95 ± 0.62^b^	2.16 ± 0.64^c^
Na (g/kg DM)	0.09 ± 0.04^a^	0.10 ± 0.03^a^	0.10 ± 0.04^a^	0.13 ± 0.04^b^
Mn (mg/kg DM)	137.66 ± 9.83^a^	142.83 ± 24.42^ab^	146.94 ± 14.73^b^	149.03 ± 11.62^b^
Fe (mg/kg DM)	309.95 ± 52.49^a^	326.45 ± 48.01^ab^	337.11 ± 45.74^b^	343.83 ± 44.99^b^
Zn (mg/kg DM)	63.85 ± 9.06^a^	67.00 ± 13.76^ab^	69.21 ± 9.06^bc^	71.88 ± 6.67^c^
Cu (mg/kg DM)	10.66 ± 1.12^a^	11.20 ± 1.47^ab^	11.64 ± 1.04^bc^	11.93 ± 1.61^c^

The same superscript letters in each row demonstrate a lack of significant difference between values (*p* < 0.05).

### Influence of the year of application on the values of the indicators studied

In the following subsection, the influence of the second factor, i.e. the year of application of L-Amino + Humus, is briefly analysed. In the analysis of variance, the p-values for years (Factor B, Tables 4 and 5) show that for several indicators the alternative hypothesis of significance of the differences between the means for each year can be accepted. However, it is worth noting that there was no evidence of an interaction between dose size and year of use (all p-values were well above 0.05), which allows the influence of each factor to be analysed separately. For all indicators for which the null hypothesis was rejected in the analysis of variance, we also analysed the mean values of all 48 samples collected each year and the percentiles of 0.1 and 0.9, as well as the corrected mean difference index between each pair of years: 1 versus 2, 2 versus 3 and 3 versus 1 (Table 9).

Most of the visual evaluation indicators show a slight average increase compared to the previous year. However, this is not the rule - leaf spot reached its highest average score in the second year of the survey. Percentiles of 0.1 reached very similar values in the first and second years of the survey, while percentiles of 0.9 reached similar values in the second and third years. The largest spread of values tended to occur in the second year of the survey. Analysis of the corrected mean difference index showed significant differences from the third year of the study for general appearance, leaf colour and stem rust, and from the first year for leaf spot (relative values Ad’ above 5%).

The mean values and tested percentiles of 0.1, 0.9 of the NDVI and SPAD indices were obtained for the second year of the study and the smallest for the third year. None of the calculated relative values of Ad’ exceeded the threshold of 5%.

The means and percentiles of the 0.1, 0.9 series of minerals showed a significant degree of irregularity. The first year was characterised by the highest values of K, Ca and Fe and the lowest values of P and Mn. The second year achieved the highest means and percentiles of P and the lowest Fe, while the third year: the highest Mn and the lowest K, Ca. The only relative value of Ad’ that exceeded the 5% threshold occurred when examining the differences in Fe concentration for the first and second years of the study.

**Table 9:** Mean values, percentiles of 0.1, 0.9 and corrected mean difference for indicators where the ‘year’ factor could be statistically significant.

	Year 1			Year 2			Year 3			A_d_’		
	pc 0.1	mean	pc 0.9	pc 0.1	mean	pc 0.9	pc 0.1	mean	pc 0.9	1 vs. 2	2 vs. 3	3 vs. 1
General aspect	5.37	7.52	8.38	5.74	7.82	8.88	7.8	8.41	8.72	-2.21%	-5.49%	10.58%
Leaf colour	5.42	7.5	8.38	5.55	7.71	9.0	7.68	8.43	9.0	-1.23%	-7.27%	11.02%
Leaf texture	5.44	7.19	8.38	4.99	7.46	8.88	6.57	7.8	8.9	-2.20%	-1.41%	5.68%
Leaf spot	5.32	7.71	9.0	6.99	8.53	9.0	7.11	8.43	9.0	-10.53%	0.24%	8.51%
Stem rust	4.81	7.73	9.0	5.48	7.86	9.0	7.91	8.73	9.0	-0.61%	-11.02%	11.46%
NDVI	0.82	0.85	0.89	0.83	0.86	0.91	0.79	0.84	0.87	-1.44%	3.28%	-1.92%
SPAD	37.01	39.7	43.83	37.75	40.51	44.37	35.89	38.84	42.92	-1.99%	4.12%	-2.19%
P	1.84	2.17	2.41	1.87	2.34	2.9	1.79	2.3	2.87	-4.93%	0.47%	2.42%
K	36.44	39.59	43.38	35.86	38.88	42.57	34.39	37.46	41.04	1.08%	2.49%	-4.97%
Ca	2.57	3.08	3.57	2.55	3.01	3.44	2.39	2.89	3.4	0.69%	2.85%	-4.71%
Mn	129.64	141.01	151.96	131.89	143.73	156.74	133.39	147.6	166.62	-1.23%	-1.73%	3.39%
Fe	306.84	340.99	378.27	267.46	320.85	375.51	276.13	326.17	378.08	5.63%	-1.40%	-4.54%

The analyses carried out suggest that the year of the study had a slight impact on the values of the indicators analysed. The changes observed in each year do not show any clear trend or regularity apart from the visual assessment, the indicators of which usually reached their highest values in the third year of the study. However, the analysis of the remaining indicators (NDVI, SPAD and mineral coefficient) makes it impossible to say which year can be considered the best or worst in terms of the parameters studied. The results suggest that the variability in the values of the indicators is due to factors other than the year of the survey.

## Discussion

The experimental agent employed in the study is L-Amino + Humus, which is distinguished by the presence of free L-α-amino acids formed through enzymatic hydrolysis. In addition to the biologically active free L-α-amino acids, L-Amino + Humus also comprises humic acids, which function as organic growth biostimulators. The application of this product to lawns is of significant benefit, as it greatly reduces the necessity for chemical treatments and intensive fertilisation. This finding is consistent with those of our previous studies^12,45^ and with the data presented in the literature^46–48^. This effect can be attributed to the alleviation of stress. For example, proline has been demonstrated to assist grasses in coping with abiotic stresses, including drought, heat and salinity^46^. Additionally, they facilitate nutrient uptake by acting as chelating agents that facilitate the uptake of essential nutrients by grass roots^47^. Furthermore, they stimulate growth by accelerating photosynthesis and growth (glycine and glutamine are precursors of chlorophyll and other important compounds)^48^. The experiment demonstrated that the application of an amino acid preparation with humic acids has a beneficial effect on the visual and functional parameters of lawns. This finding corroborates the synergistic effect of humic acids and amino acids as biostimulants. Similar effects have been documented in the scientific literature^48–50^. The combined application of these biostimulants has been demonstrated to enhance plant growth and stress tolerance through a range of mechanisms, including optimised nutrient uptake, elevated physiological activity and improved soil structure^51,52^. A statistically significant increase was observed in these parameters with the highest dose of the experimental agent (3 L ha^−1^) (Table 4). The beneficial impact of the applied fertiliser can be attributed to its diverse composition. The formulation contains humic compounds, which are naturally occurring components of soil organic matter. These compounds are formed through the decomposition of plant, animal and microbial residues, as well as through the metabolic activity of soil microorganisms. These compounds have been demonstrated to exert a beneficial effect on plant growth. The amino acids present in L-Amino + Humus are instrumental in numerous physiological processes of plants. They are indispensable for plant growth and development, as well as for the regulation of intracellular pH and metabolic energy production. Furthermore, amino acids enhance plant resilience to abiotic (e.g. drought, extreme temperatures) and biotic (e.g. pathogens, pests) stresses. The presence of humic acid in the formulation has been demonstrated to enhance soil structure, augment nutrient availability and facilitate root system development, which collectively contribute to an improved lawn condition and appearance.

The aforementioned properties of L-Amino + Humus render it an efficacious means of improving lawns while reducing the necessity for intensive chemical fertilisation. The popularity of biostimulants, such as this formulation, is increasing due to their favourable ecological impact and efficacy in green care.

This may be attributed to the role of humic acid in increasing the availability of nutrients in the soil by influencing the activity of soil microorganisms. Furthermore, humic acid contains NPK (nitrogen, phosphorus and potassium) and some micronutrients essential for optimal plant growth^53^. Amino acids have been demonstrated to enhance the resilience of the plant’s immune system, thereby reducing the adverse impact of disease. Furthermore, they may contribute to the regulation of hormonal control within plants, which in turn facilitates vegetative and root growth^54^.

These findings are in accordance with those previously documented by Canaway^55^ in relation to *Lolium perenne* cv. Loretta, Hunter and Butler^56^ on *Agrostis stolonifera*, El-Sayed et al.^57^ on Tifway Bermudagrass, and Bettoni et al.^58^ on Kalanchoe.

In a field experiment conducted at a nursery (Hort. Res. Inst., ARC, in Giza, Egypt) during the 2014 and 2015 seasons^20^. The effect of spraying humic acid at concentrations of 0, 5 and 10 ml/L, and adding a mixture of amino acids as soil irrigation at rates of 0, 1 and 2 g/pot, individually or in combinations, on the growth, cover index (%), and chemical composition of seagrass (*Paspalum vaginatum*, Swartz.) grown in 40 cm diameter plastic pots filled with sand was investigated. The results demonstrated that all treatments resulted in improvements in plant height, cover index, number of plants per pot, and fresh and dry green matter in comparison to the control^20^. Furthermore, the chemical analysis revealed an enhancement in the chlorophyll a, b and carotenoid content in the leaves and total sugars in the green matter. The most optimal outcome was achieved through a combination of humic acid at a concentration of 10 ml/l and a mixture of amino acids at a dose of 2 g/pot, which exhibited the highest mean values.

A number of studies^2,59^ have shown positive effects of various amino acid-containing biostimulants on lawn quality. Kamyab et al.^60^ showed that humic acid has a positive effect on the morphological and biochemical characteristics of common turf grasses.

The commercial preparation utilized in this study comprises humic compounds and sterols, in addition to free amino acids. The formation of diverse aggregates and micelles in such a mixture has the potential to enhance the solubility of specific chemical compounds. The formation of inverted phospholipid micelles has been demonstrated^61^, to enhance the solubility of a range of inorganic and organic compounds, as well as amino acids and smaller peptides. It has been demonstrated that humic compounds possess the capacity to form macromolecular structures^62^, frequently comprising a multitude of low-molecular compounds, including amino acids, within these aggregates. Analogous studies^2^ utilising a biostimulant from the same manufacturer, comprising solely amino acids, yielded analogous results with amino acid concentrations twice those employed in the present study. This also corroborates the hypothesis that additives such as humic acids, sterols and lipid compounds exert a synergistic effect.

The preparation based on amino acids and humic acids significantly improves the qualitative value of turf, enhancing visual, functional, and chemical parameters. The formulation improves overall appearance, turf density, leaf color and texture, and disease resistance, mainly by stimulating root development, chlorophyll content, and plant defense mechanisms. These results are consistent with those of Zhang et al.^28^ and Liu et al.^27^ and highlight the positive effects of the formulation on turf quality. The authors suggest that the amino acids in the formulation promoted abiotic stress tolerance through osmotic and hormonal regulation, while the humates improved soil structure and the ability of roots to absorb mineral nutrients. The results are consistent with the literature which suggests that amino acids and humic acids have a synergistic effect, improving both functional and visual aspects of turf^28,63^. The analyses also suggest that the year of the study had little effect on the indicators studied. While visual assessment scores tended to peak in the third year, no clear trends emerged for other parameters. The annual fluctuations in these indicators do not demonstrate a consistent pattern, suggesting that their variability is likely attributable to factors external to the survey period itself.

## Conclusions

The foliar application of the amino acid-humic acid biostimulant (L-Amino+^®^ Humus) significantly enhanced both the functional and aesthetic quality of turfgrass. The most pronounced effects were observed at the highest dose (3.0 L ha^−1^, Variant IV), which elicited statistically superior results across key parameters compared to untreated controls. Notably, this dose reduced snow mould (*Microdochium nivale*) incidence by 8% and brown spot (*Rhizoctonia solani*) prevalence by 12%, while simultaneously elevating NDVI and SPAD values by 6% and 8%, respectively. These improvements reflect enhanced photosynthetic efficiency and chlorophyll content, corroborating the biostimulant’s role in bolstering plant vitality.

Dose II (2.0 L ha^−1^) also demonstrated efficacy in improving canopy density and overall visual appeal, though its effects were less consistent than those of the highest dose. Importantly, the biostimulant facilitated greater macro- and micronutrient assimilation (e.g., nitrogen, iron), underscoring its utility in sustainable nutrient management.

While the study confirmed the dose-dependent benefits of L-Amino+^®^ Humus, interannual variability in results was minimal. Visual assessment scores peaked in the third year, but trends in NDVI, SPAD, and mineral coefficients lacked clear temporal patterns, suggesting that external factors (e.g., microclimate, soil dynamics) exerted greater influence than the study’s chronological progression.

Key Recommendations and Implications:

- Agricultural Practice: Apply L-Amino+^®^ Humus at 3.0 L ha^−1^ to optimise turfgrass quality, disease resistance, and nutrient uptake.
- Sustainability: The formulation offers an eco-functional alternative to conventional fertilisers by enhancing stress resilience and reducing dependency on synthetic inputs.
- Further Research: Investigate dose optimisation across diverse plant species and environmental contexts to refine application guidelines.

In summary, this work validates the synergistic role of amino acids and humic acids in turfgrass biostimulation, aligning with existing literature on their combined physiological benefits. By integrating visual, metabolic, and ecological advantages, the preparation represents a strategic tool for modern, sustainable horticulture.

## Electronic supplementary material

Below is the link to the electronic supplementary material.

Supplementary Material 1

## Data Availability

The datasets used and/or analysed during the current study available from the corresponding author on reasonable request.
