# Supplementary material for: The impact of an amino acid-humus preparation on lawn boning value
Source: Sci Rep. 2025 Feb 24;15:6607. doi: 10.1038/s41598-025-90862-y (PMC11850864; doi:10.1038/s41598-025-90862-y)
Supplement: Supplementary file 1 — Supplementary Material 1 [file 41598_2025_90862_MOESM1_ESM.docx]

**The Impact of an Amino Acid-Humus Preparation on Lawn Boning Value**

Adam Radkowski^1^, Iwona Radkowska^2^, Karen Khachatryan^3^, Michał Kozdęba^4^, Henryk Bujak^5,6^ and Karol Wolski^7^

^1^ Department of Agroecology and Plant Production, University of Agriculture in Kraków, Mickiewicza 21, 31-120 Kraków, Poland; ORCID: 0000-0002-3146-6212; adam.radkowski@urk.edu.pl

^2^ Department of Cattle Breeding, National Research Institute of Animal Production, 32-083 Balice, Krakowska 1, Poland; ORCID: 0000-0002-8780-1585; iwona.radkowska@izoo.krakow.pl

^3^ Laboratory of Nanomaterials and Nanotechnology, Faculty of Food Technology, University of Agriculture in Krakow, Balicka 122, 30-149 Krakow, Poland;
ORCID: 0000-0001-7823-5406; karen.khachatryan@urk.edu.pl

^4^Department of Production and Power Engineering, University of Agriculture in Krakow, ul. Balicka 116 b, 30-149 Krakow, Poland; ORCID: 0000-0002-3167-2606; michal.kozdeba@urk.edu.pl

^5^ Department of Genetics, Plant Breeding and Seed Production, Wrocław University of Environmental and Life Sciences, Grunwaldzki 24A, 53-363 Wrocław, Poland; ORCID: 0000-0001-8095-2105; henryk.bujak@upwr.edu.pl

^6^ Research Center for Cultivar Testing (COBORU), 63-022 SłupiaWielka, Poland; ORCID: 0000-0001-8095-2105; h.bujak@coboru.gov.pl

^7^ Department of Agroecology and Plant Production, Wrocław University of Environmental and Life Sciences, Grunwaldzki 24A, 50-363 Wrocław, Poland; ORCID: 0000-0001-9324-4968; karol.wolski@upwr.edu.pl

***** Correspondence: karen.khachatryan@urk.edu.pl; Tel. +48-126624846

**Supplementary material**


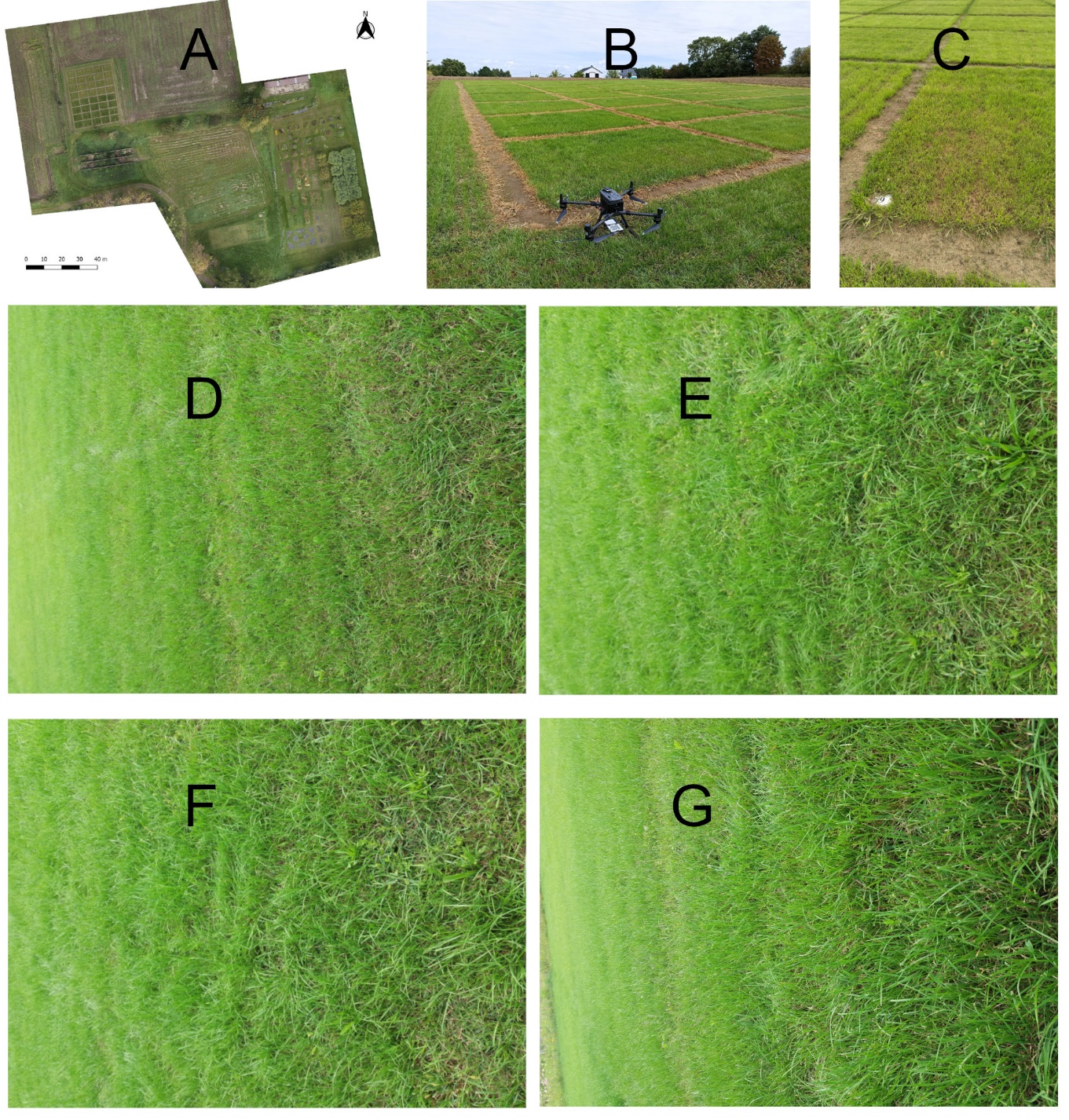


**Figure S1.** Location of the experiment and appearance of the selected turfgrasses. A _ View from the drone, B and C _ General view, D _ Variant I, E _ Variant II, F _ Variant III and G _ Variant IV.

Below are basic plots of all the data obtained. Four samples were analysed in each season (spring, summer, autumn) for each of the four variants (var I, var II, var III, var IV). The graphs show the average values of these samples, and in the legend (av) the average value obtained over the entire study period, i.e. three years, has been added. Line graphs have been used to better illustrate the upward and downward trends of the different variants.


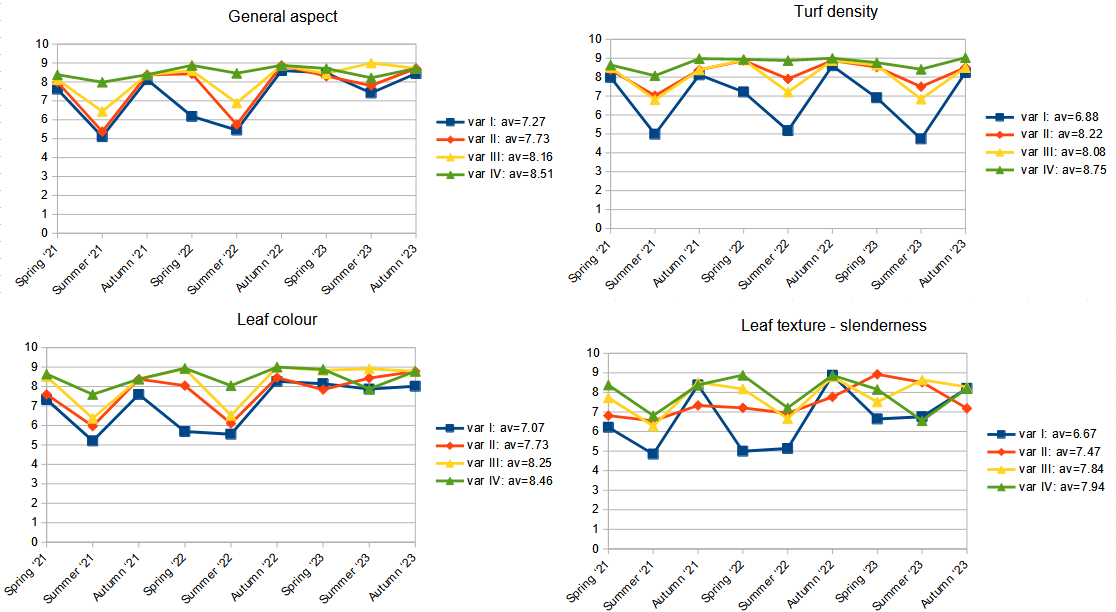


**Figure S2.** Visual assessment indices for variants I (var I), II (var II), III (var III) and IV (var IV).


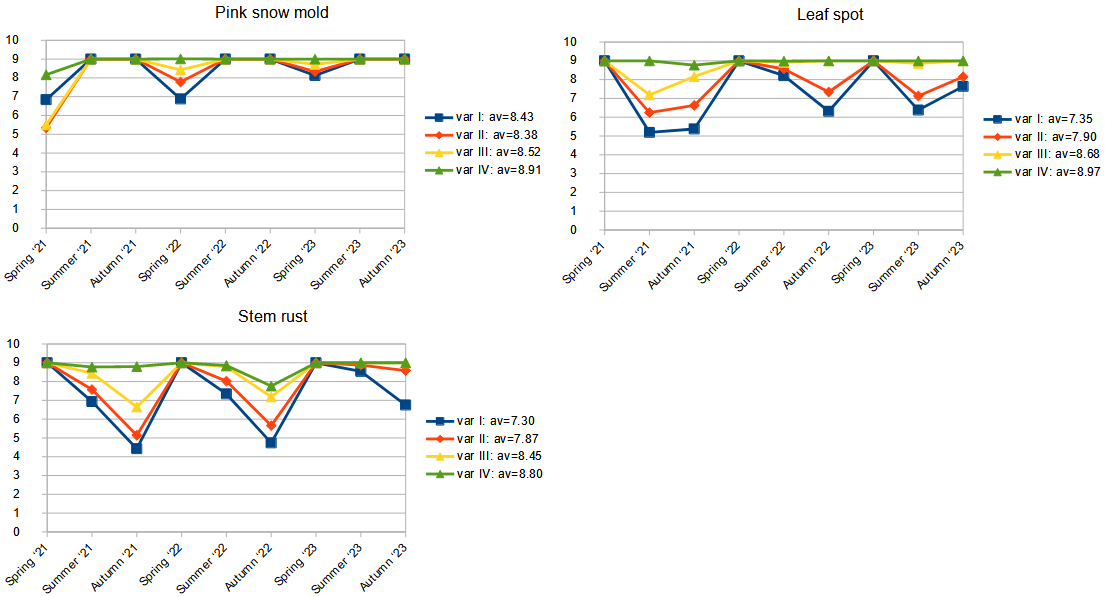


**Figure S3.** Disease susceptibility indices for variants I (var I), II (var II), III (var III) and IV (var IV).


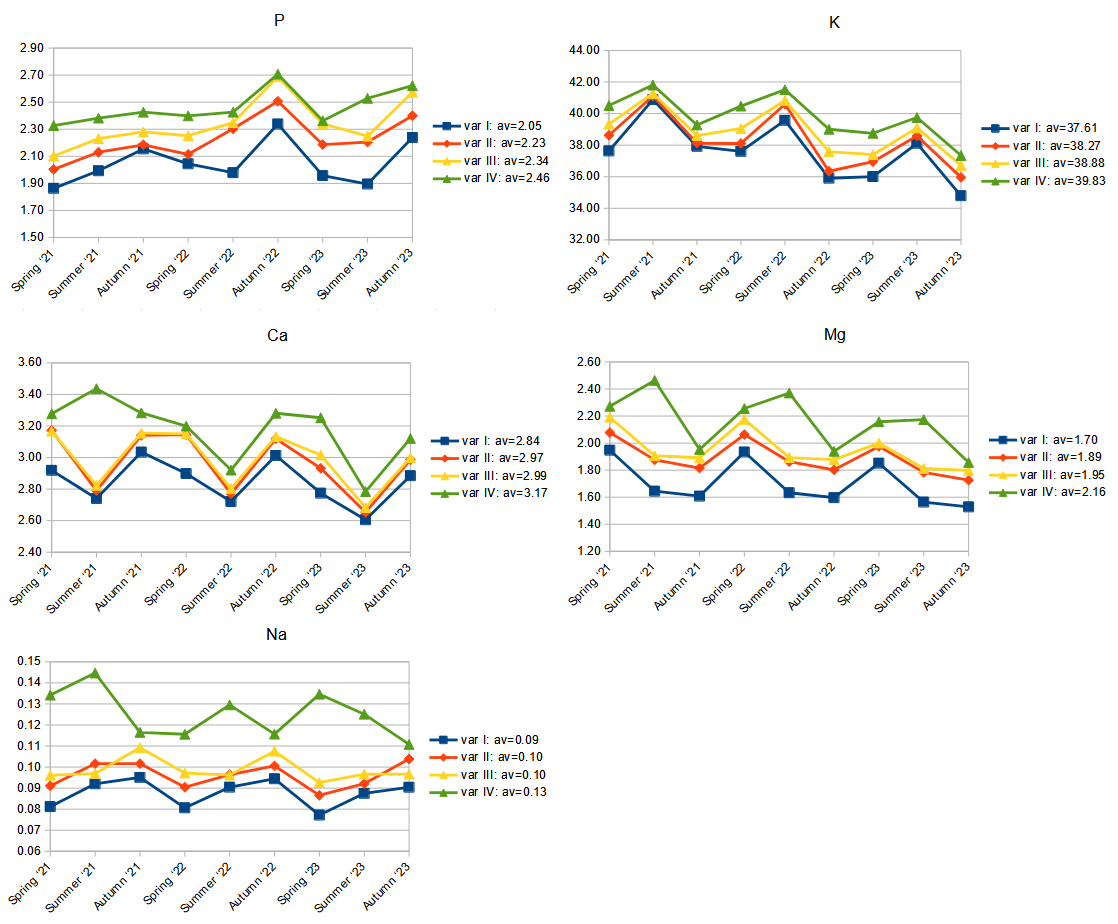


**Figure S4.** Macronutrient indices for variants I (var I), II (var II), III (var III) and IV (var IV) in g·kg^-1^DM.


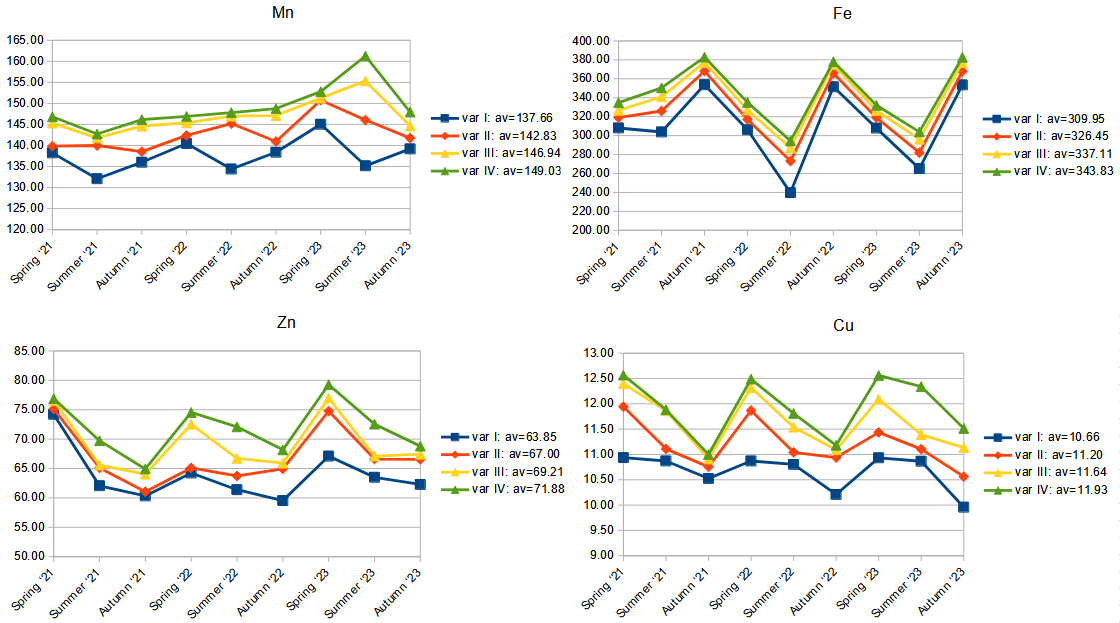


**Figure S5.** Micronutrient indices for variants I (var I), II (var II), III (var III) and IV (var IV) in mg·kg^-1^DM.
